# Racismo ambiental, mineração e saúde mental da população negra

**DOI:** 10.1590/0102-311XPT045324

**Published:** 2024-07-29

**Authors:** Ivison Luan Ferreira Araújo, Ana Cecilia Rodrigues dos Santos Godoi, Luiz Paulo Ribeiro

**Affiliations:** 1 Universidade Federal de Minas Gerais, Belo Horizonte, Brasil.; 2 Associação Estadual de Defesa Ambiental e Social, Belo Horizonte, Brasil.

O mais recente livro ^1^ acerca do racismo ambiental é organizado por Mariana Belmont - jornalista, articuladora, comunicadora, ativista, de movimentos ambientalistas e periféricos -, sob responsabilidade do Instituto de Referência Negra Peregum. O livro *Racismo Ambiental e Emergências Climáticas no Brasil* tem início com um convite para a reflexão, desvelando no racismo ambiental a exploração intensiva da vida e dos recursos naturais, que é distribuído territorialmente de forma desigual, onerando principalmente a população negra e sua relação com as lutas, a cura e a história ancestral das religiões de matrizes africanas, da população negra e periférica no Brasil. Nessa introdução, ressalta-se a importância de unir o passado com o futuro como estratégia de resistência diária e de transformação dos sujeitos para a construção de uma sociedade antirracista.

O racismo ambiental é apresentado como uma forma contemporânea de manifestação do racismo e da discriminação racial que marca a vida das pessoas em seus territórios. Além da dinâmica climática e formas de atualização do racismo, é apresentada a compreensão sobre o papel que o Estado exerce na sociedade brasileira e nos espaços decisórios em relação às emergências climáticas, problematizando o termo justiça ambiental como forma de invisibilizar o fundamento racial, combinado aos fatores econômicos e de gênero da crise ambiental e climática e das possíveis soluções para o quadro de calamidade mundial. No Brasil, em relação à mineração e aos últimos rompimentos de barragem no Estado de Minas Gerais - ocasionados pelas mineradoras Samarco e Vale S/A, em Mariana e Brumadinho, respectivamente -, é ressaltada a importância de que precisam ser considerados veredictos de racismo ambiental e não apenas acidentes ^1^. Na mesma linha de raciocínio, é preciso evidenciar quais interesses, grupos e forças socioeconômicas que provocam crimes ambientais, gerando violências e vulnerabilidades para as populações ribeirinhas, indígenas e afrodescendentes, que já são historicamente violentadas, empobrecidas e vulnerabilizadas.

Fundamentada em sua argumentação e na potencialidade de sua análise, Mariana Belmont aborda o racismo ambiental a partir de questionamentos, como “*Qual a cor dos corpos levados pelas enchentes, soterrados pelos deslizamentos e que são afetados pela escassez de alimentos nas cidades?*” (p. 17). Diante dessa mobilização, podem ser feitas também as perguntas: quais são os corpos atingidos pelos grandes empreendimentos? Qual a relação entre o racismo ambiental e a saúde mental?

Isso porque, no final de 2015 e no início de 2019, ocorreram em Minas Gerais dois desastres/crimes sociotecnológicos, sendo considerados os mais graves desastres envolvendo barragens de mineração do século XXI no Brasil, causando rupturas e interrupções no cotidiano do território, além de grandes perdas e danos, inclusive relacionados à saúde, que ultrapassam as capacidades de resposta das comunidades, municípios e regiões atingidas ^2^ ^,^ ^3^.

Segundo o *Censo Demográfico* de 2022 do Instituto Brasileiro de Geografia e Estatística (IBGE) ^4^, os 27 municípios atingidos pelo desastre/crime sociotecnológico em Brumadinho contêm uma população geral majoritariamente negra (parda e preta), perfazendo aproximadamente 68% da população residente. Entretanto, quando olhado com profundidade a partir do contexto de rompimento, a porcentagem pode aumentar. A organizadora do livro cita que no rompimento de Mariana em 2015 teve, entre a população atingida, a porcentagem de 84% de negros, indo, dessa forma, ao encontro do racismo ambiental evidenciado na obra.

Ainda na introdução, é citada a Conferência Mundial contra o Racismo, a Discriminação Racial, a Xenofobia e Formas Correlatas de Intolerância, sediada em Durban (África do Sul) no ano 2001. Espaço que, já àquela época, fez referência ao meio ambiente e sua relação direta com a saúde das populações negras e ao processo de tomada de decisão. Contribuindo com a compreensão do livro, entende-se que os potenciais efeitos desses desastres para a saúde, são inúmeros a curto, médio e longo prazos ^5^. Sendo assim, o Ministério da Saúde, a Fundação Oswaldo Cruz, em Minas Gerais, e a Universidade Federal do Rio de Janeiro desenvolvem estudos para avaliar as condições de vida e saúde dos atingidos(as) de Brumadinho ^6^.

Esses estudos demonstram sinais de modificações na vida e na saúde da população atingida, como no inquérito realizado em Barra Longa (Mariana) em 2017, no qual 43,5% dos(as) entrevistados(as) relataram ter problemas de saúde após o rompimento, sendo um dos principais problemas os quadros depressivos ^7^. Em Brumadinho, existe um impacto psicossocial que pode agravar o quadro de doenças crônicas e aumentar a ocorrência de transtornos mentais, como depressão e ansiedade ^8^.

Já a Matriz de Medidas Reparatórias Emergenciais, de Brumadinho, construída a partir dos espaços participativos com os(as) atingidos(as) em 2021 junto com assessoria técnica independente, apresenta aspectos e estudos que identificam o aumento considerável de doenças mentais e físicas na população, ocasionando agravamentos e desenvolvimento de transtornos mentais, sofrimento, aumento do uso e abuso de drogas lícitas e ilícitas, tentativas e suicídios e aumento da violência doméstica ^9^.

Por sua vez, o Projeto Saúde Brumadinho menciona aspectos importantes sobre o predomínio de sintomas psiquiátricos e seus fatores associados, além do uso de medicamentos psicotrópicos pela população local. É importante destacar algumas questões sobre o estudo: prevalência de sintomas psiquiátricos e seus fatores associados na população adulta da área atingida pelo rompimento da barragem de rejeitos, em que 56,7% das pessoas entrevistadas são mulheres e 57,7% são pessoas negras. Entre os relatos, os sintomas depressivos foram de 29,3%, os de transtorno de estresse pós-traumático (TEPT) foram de 22,9% e a presença de ideação suicida ou automutilação 12,6% ^10^.

Os sintomas de TEPT, depressivos, ansiosos, ideias de morte/automutilação e piora da qualidade de sono estão manifestos em pessoas entre 18-59 anos, Mulheres, negras, não casadas, com Ensino Médio e residentes em regiões de mineração. Ressalta-se que, para os casos de ideias de morte/automutilação, a escolaridade muda para o Ensino Fundamental. O estudo ainda afirma que diferentes sintomas psiquiátricos encontrados em Brumadinho estão acima da média percentual verificada na população brasileira ^10^. Entende-se, dessa forma, que existe uma relação entre o racismo ambiental, o rompimento da barragem e com o adoecimento mental da população atingida, onerando com agravos e de forma massiva a população negra e de mulheres.

Na segunda parte do livro, a organizadora detém-se sobre algumas temáticas que reforçam a defesa sobre o processo de racismo ambiental no Brasil, ao exemplificar como a epidemia do Zika vírus teve questões raciais estruturantes como um dos principais fatores de proliferação, entendendo que a população negra tem vulnerabilidades estruturais, como moradia, acesso a saneamento básico, água potável e socioeconômicas, processos que excluem, exploram e dominam essa população a partir da articulação das desigualdades sociais e que desembocam no processo saúde-doença ^1^.

Com todo esse enquadramento histórico, social e contextual, percebe-se que existe uma multiplicidade de situações, exposições, riscos e efeitos sobre a saúde, em específico à saúde mental, da população negra a partir de um rompimento de barragem, uma grande chuva ou epidemia, por exemplo, que é mascarada e agravada pela profunda desigualdade social e pelo racismo institucionalizado no Brasil ^11^. Entende-se, assim, que o conceito de racismo ambiental é um conceito promissor para a saúde pública, necessitando de novas articulações com a políticas existentes no Sistema Único de Saúde e outras mais que consigam ser efetivas para a diminuição dos agravos e para a coexistência e a resiliência de territórios saudáveis no Brasil.

## References

[1] Belmont M (2023). Racismo ambiental e emergências climáticas no Brasil.

[2] Freitas CM, Barcellos C, Asmus CIRF, Silva MA, Xavier DR (2019). Da Samarco em Mariana à Vale em Brumadinho desastres em barragens de mineração e Saúde Coletiva. Cad Saúde Pública.

[3] Carneiro KG, Souza TR Anais do 17º Encontro Nacional da Associação de Pós-graduação e Pesquisa em Planejamento Urbano e Regional..

[4] Instituto Brasileiro de Geografia e Estatística (2023). Censo Demográfico 2022.

[5] Silva EL, Silva MA (2020). Segurança de barragens e os riscos à saúde pública. Saúde Debate.

[6] Fundação Oswaldo Cruz Projeto Saúde Brumadinho..

[7] Vormittag EMPAA, Oliveira MA, Rodrigues CG, Gleriano JS (2017). Avaliação dos riscos em saúde da população de Barra Longa/MG afetada pelo desastre..

[8] Barcellos C (2019). Avaliação dos impactos do desastre de Brumadinho sobre a saúde..

[9] Associação Estadual de Defesa Ambiental e Social (2021). Matriz de medidas reparatórias emergenciais..

[10] Garcia FD, Neves MCL, Firmo JOA, Peixoto SV, Castro-Costa E (2022). Prevalência de sintomas psiquiátricos e seus fatores associados na população adulta da área atingida pelo rompimento da barragem de rejeitos: Projeto Saúde Brumadinho.. Rev Bras Epidemiol.

[11] Herculano S (2008). O clamor por justiça ambiental e contra o racismo ambiental. InterfacEHS.

